# Supplementary material for: Energy evolution mechanism of structural surfaces in sandstones with different dips based on the energy principle
Source: PLoS One. 2024 Mar 21;19(3):e0300931. doi: 10.1371/journal.pone.0300931 (PMC10956845; doi:10.1371/journal.pone.0300931)
Supplement: Attachment — Submitted filename: Response to Reviewers.docx [file pone.0300931.s002.docx]

Dear Editors and Reviewers:

Thank you for your letter and the reviewers’ comments concerning our manuscript entitled “Energy evolution mechanism of structural surfaces in sandstones with different dips based on the energy principle” (ID: PONE-D-24-01554). Those comments are all valuable and very helpful for revising and improving our paper and the important guiding significance of our research. We have studied the comments carefully and tried our best to revise and improve the manuscript according to the constructive comments. We appreciate for Editors/Reviewers' warm work earnestly. We would be glad to respond to any further questions and comments that you may have. All the revised portions are marked in red in the article. The main corrections in the paper and the responses to the reviewers' comments are as follows:

- **Reviewer #1:**

**1. Comment:** 1. Most of the research results of scholars listed in the introductory part of the paper are the results of mechanical tests on rock bodies containing structural surfaces, and there are relatively few relevant results involving energy evolution; in addition, the relevant results of the principle of minimum energy consumption also need to be listed.

**Response:** Thanks for reviewer’s comment. The authors have already listed energy evolution and minimum energy consumption-related results in the introduction.

**2. Comment:** In Section 2.2, the author should describe in detail the source of rock specimens.

**Response:** Thanks for reviewer’s comment. The authors have described in detail the origin of the rock specimens.

**3. Comment:** The colors of the stress-strain curves in Fig. 8 for plots a, b, c, and d are not uniform. It is recommended that the authors re-plot the curves in the text.

**Response:** Thanks for reviewer’s comment. The different colored stress-strain curves in Fig. 8 correspond to Fig. 3, but this does detract from the aesthetics and I have standardized them.

**4. Comment:** Some references are not cited correctly, and the writing format and English expression should follow the standards of the International Journal.

**Response:** Thanks for reviewer’s comment. The author has revised it carefully.

**5. Comment:** The language of this manuscript is not high enough and there are many unclear expressions and grammatical errors. Authors should improve the quality of the language.

**Response:** Thanks for reviewer’s comment. The manuscript has been referred to professionals for touch-ups and various inappropriate expressions and grammatical issues have been corrected.

- **Reviewer #2:**

**1. Comment:** Please add the line number.

**Response:** Thanks for reviewer’s comment. The author has added line numbers to the text.

**2. Comment:** The objectives and the rationale of the study should be clearly stated.

**Response:** Thanks for reviewer’s comment. The authors have stated at the end of the abstract.

**3. Comment:** The background of the problem can be presented in the introduction.

**Response:** Thanks for reviewer’s comment. The authors have provided an introduction.

**4. Comment:** Would you mind providing an explanation for Ue and Ud in Figure 7? Additionally, could you specify the citation for Figure 7 in the manuscript?

**Response:** Thanks for reviewer’s comment. Consider a rock unit deformed by an external force, assuming that there is no heat exchange between the physical process and the outside world, i.e., a closed system and that the total input energy from the external work is U. From the first law of thermodynamics, we have U = Ud + Ue, where Ud is the dissipated energy and Ue is the releasable elastic strain energy. The authors have already explained Ue and Ud in the text and pointed out the references to Fig. 7.

**5. Comment:** How was equation 1 derived, and could you please provide a more detailed explanation for its derivation?

**Response:** Thanks for reviewer’s comment. As shown in Figures 4, 5, and 6, due to the good fit of the experimental data, the fitted three curve equations derived from Origin were then linked as a function of peak strain, compressive strength, and modulus of elasticity concerning the inclination angle, and a, b, c, d, f, and g were used as the fitted parameters in place of the specific values in the equations, respectively.

**6. Comment:** How many rock samples have you analyzed?

Response: Thanks for reviewer’s comment. The rock samples were all cut from the sandstone blocks of the Anjialing North Gang slope of China Coal Pingshuo, and 3 rock samples were processed for each inclination, totaling 12 rock samples, and due to the influence of space, only the most representative group was selected and put into the text.

**7. Comment:** The damage threshold of the rock sample is only related to its compressive strength, peak strain, and elastic modulus, and is a comprehensive index of all three”. You mentioned the term 'only' in the conclusion. Could you elaborate on how you arrived at these conclusions?

**Response:** Thanks for reviewer’s comment. As can be seen from the derived Eq. (23), the only parameters that affect the damage threshold in this paper are compressive strength, peak strain, and modulus of elasticity, but the word "only" has been deleted from the manuscript because it is indeed too absolute.

**8. Comment:** In the process of deep mining, appropriate protective measures shall be taken for rock bodies containing structural surfaces with different inclinations, in order to prevent geological hazards such as collapses and sheet gangs from occurring in the rock bodies.” The stress state in deep mining is complex, and relying solely on uniaxial compressive tests may be insufficient. How did you come to this conclusion?

**Response:** Thanks for reviewer’s comment. In the early review of the literature, the authors found that many scholars for the deep rock body force analysis also from the uniaxial compression experiment, but as you said, the deep mining stress state is complex, only uniaxial compression test is not enough, so the authors of the original conclusion were modified, and will be used in the subsequent study of triaxial compression test to continue to analyze the mechanical properties of the structural surface of the different inclination angle.

**9. Comment:** Most of the references are relatively outdated, and some of the latest research related to rock mechanisms can be added, such as 10.1016/j.fuel.2023.129584, 10.1016/j.compgeo.2024.106095.

**Response:** Thanks for reviewer’s comment. The authors have added relevant and up-to-date research findings.

**10. Comment:** The abstract is lengthy; kindly condense it.

**Response:** Thanks for reviewer’s comment. The authors have streamlined some of the abstracts.
